# Anti-Neuroinflammatory Potential of Natural Products in the Treatment of Alzheimer’s Disease

**DOI:** 10.3390/molecules28031486

**Published:** 2023-02-03

**Authors:** Mingzhenlong Deng, Wanli Yan, Zhicheng Gu, Yan Li, Lei Chen, Bin He

**Affiliations:** State Key Laboratory of Functions and Applications of Medicinal Plants, Engineering Research Center for the Development and Application of Ethnic Medicine and TCM (Ministry of Education), Guizhou Provincial Key Laboratory of Pharmaceutics, School of Pharmacy, School of Basic Medical Science, Guizhou Medical University, Guiyang 550004, China

**Keywords:** Alzheimer’s disease, neuroinflammation, natural products, anti-AD

## Abstract

Alzheimer’s disease (AD) is an age-related chronic progressive neurodegenerative disease, which is the main cause of dementia in the elderly. Much evidence shows that the onset and late symptoms of AD are caused by multiple factors. Among them, aging is the main factor in the pathogenesis of AD, and the most important risk factor for AD is neuroinflammation. So far, there is no cure for AD, but the relationship between neuroinflammation and AD may provide a new strategy for the treatment of AD. We herein discussed the main etiology hypothesis of AD and the role of neuroinflammation in AD, as well as anti-inflammatory natural products with the potential to prevent and alleviate AD symptoms, including alkaloids, steroids, terpenoids, flavonoids and polyphenols, which are available with great potential for the development of anti-AD drugs.

## 1. Introduction

Alzheimer’s disease (AD) was discovered by Dr. Alois Alzheimer in 1906 [1]. It is an age-related chronic irreversible neurodegenerative disease and the main cause of senile dementia [1,2]. Patients with advanced AD have physiological and psychological problems, cannot live normally and eventually die of complications such as infection [2,3]. Age growth is the most important risk factor for AD [4]. With the aging of the population, the number of AD patients will increase dramatically. At present, nearly 50 million people worldwide suffer from dementia, which is expected to reach 150.11 million in 2050 [5,6,7]. From the perspective of economic cost, AD has become an important public health problem with an estimated cost of USD 1.1 trillion by 2050 [5]. The dramatic increase in the number of patients and socio-economic costs points to the urgent need for effective preclinical prevention of AD and the development of new drugs to stop the disease from progressing [8]. The neuropathological characteristics of AD are β-Amyloid protein (Aβ) aggregation to form plaques, hyperphosphorylated neurofibrillary tangles (NFT) of tau protein and extensive neuronal loss [9,10]. Studies have shown that Aβ and NFT can induce chronic and persistent neuroinflammation in AD patients, which is characterized by activation of macrophages in the brain, induction of excessive secretion of pro-inflammatory cytokines and chemokines such as interleukin-1β (IL-1β), interleukin-6 (IL-6) and tumor necrosis factor [7]. The chronic and persistent neuroinflammation in turn can promote the production of Aβ and NFT, further leading to neurotoxicity and apoptosis [11]. These pathological states form a vicious circle [12].

Although great efforts in the research and development of AD therapeutic drugs have been made, many disease-modifying drugs (DMF) have failed in clinical trials due to the complex pathological nature of the disease, severe side effects of drugs and poor permeability of the blood–brain barrier (BBB) [13]. Since 2003, only Aducanumab has been approved by the US Food and Drug Administration (FDA) for the treatment of AD in June 2021 [14,15,16]. Previously, the drugs for the treatment of AD approved by FDA include cholinergic inhibitors (rivastigmine, donepezil, galantamine) and an N-methyl-D-aspartate (NMDA) receptor antagonist (memantine) [17,18]. However, these drugs can only delay the progress of AD and relieve symptoms, and there is no effective treatment to cure this disease yet.

Therefore, the development of new drugs for the treatment of AD is still an important task for medicinal chemists. At present, the development of AD drugs is diversified. On the one hand, more new treatment methods, such as gene therapy and enzyme therapy, are mostly in preclinical stages, while small molecules are in all stages of development by continuing to focus on classic targets such as Aβ and tau [19]. On the other hand, there are emerging future theories for the development of new therapies for AD such as disease modification therapy (DMT), molecular chaperones and natural products [20]. Natural products have attracted more and more attention, since natural products with anti-inflammatory activity may have certain therapeutic potentials for treating AD [7,8,21,22,23,24,25].

## 2. Pathological Hypothesis of Alzheimer’s Disease

**Figure 1:**
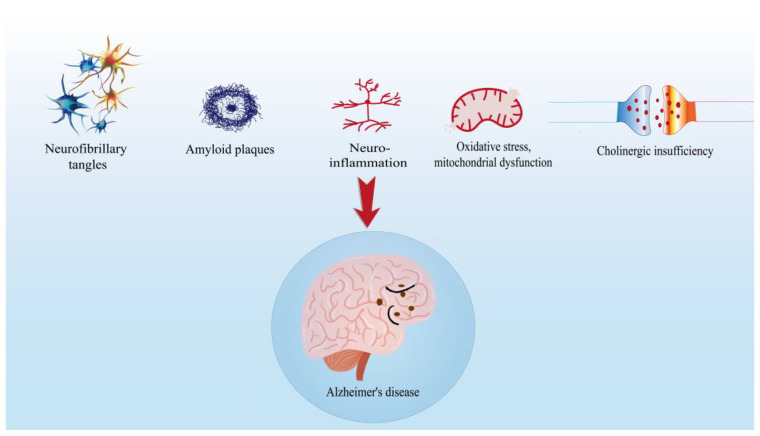
The etiology hypothesis of Alzheimer’s disease.

Extensive studies have been performed to investigate the pathogenesis of AD; however, the complex nature of AD and its psychological and physiological complications has become a huge obstacle to elucidate the pathogenesis [5,26]. At present, the exact pathogenesis of AD is not completely clear. The etiology hypothesis of AD mainly includes cholinergic hypothesis, β-Amyloid protein hypothesis, tau protein hypothesis, oxidative stress and neuroinflammation hypothesis (Figure 1) [21,27,28,29,30].

### 2.1. Cholinergic Hypothesis of Alzheimer’s Disease

Acetylcholine (ACh), the cholinergic neurotransmitter, is the most important neurotransmitter in the brain and directly participates in several physiological activities such as memory, attention, learning and other cognitive functions [31]. Increased clinical symptoms in AD patients are associated with reduced ACh-mediated neurotransmission in the cerebral cortex and hippocampus [5]. During AD, the loss of cholinergic neurons in the basal forebrain occurs because of a decrease in the activity of acetylcholine transferase (ChAT), which is responsible for the synthesis of ACh. In the meantime, the activities of acetylcholinesterase (AChE) and butyrylcholinesterase (BChE) both are increased, which may be highly related to the fact that the deposition of Aβ causes a reduction in choline uptake and a release of ACh [32,33,34]. The increase in AChE and BChE activity and the decrease in ChAT activity led to the decrease in ACh level in AD patients [2,35,36]. With the progression of AD, it is observed that the neurotransmitter is lost while signal transmission is terminated, eventually leading to cognitive and memory dysfunction [37].

### 2.2. Amyloid Hypothesis of Alzheimer’s Disease

Amyloid precursor protein (APP) is a transmembrane protein widely present in the brain [2,38]. It is mainly localized in the synapses of neurons and plays a role in neurite growth and synaptogenesis, transmembrane signal transduction, cell adhesion, etc. [39]. Aβ is a neurotoxic protein, which is produced by the cleavage of APP via β-secretase and γ-secretase [40]. Aβ usually exists in various forms of monomers, oligomers, polymers and insoluble fibrous aggregates. Additionally, soluble Aβ monomer and other Aβ monomer interactions gradually transform into oligomers, polymers and fibrous aggregates [41]. Aβ fiber aggregates further form Aβ plaques, which first accumulate in the cerebral cortex and then expand to other brain regions with the end-stage pathology, such as the hippocampus and basal forebrain [42]. This significant feature can be observed in the brain of AD patients [2,43]. The accumulation of Aβ induces neuroinflammation and triggers a neurodegenerative cascade, neurofibrillary tangles and ultimately leads to neuronal loss in the affected area of the brain [39].

### 2.3. Tau Protein Hypothesis of Alzheimer’s Disease

Tau is a soluble microtubule-associated protein (MAP) [44,45]. Tau phosphorylation in the N-terminal region (such as Ser46, Thr123, Ser198), the repeat region (Ser262 and Ser356) and the C-terminal region (such as Ser396, Ser400, Thr403) is controlled by a variety of enzymes including A kinase, C kinase, cyclin-dependent kinase 5 (CDK-5), glycogen synthase kinase 3β (GSK-3β) and mitogen-activated protein kinase (MAPK) [46,47]. The degree of tau phosphorylation in a normal brain is low while the hyperphosphorylation of tau is found in the brain of AD patients [45,48]. This abnormal phosphorylation is usually caused by Aβ and neuroinflammation [5]. Aβ induces tau hyperphosphorylation by enhancing the activity of GSK-3β and CDK-5 [46]. Abnormally phosphorylated tau is converted into paired helical filaments (PHF) and aggregated to form NFT [44,45,48]. Hyperphosphorylated tau induces neuronal apoptosis via activation of receptor interacting protein kinase 1, receptor interacting protein kinase 3 and mixed lineage kinase domain-like pseudokinase (RIPK1/RIPK3/MLKL) and nuclear factor kappa-B (NF-κB) pathway-mediated necroptosis and inflammation [49]. 

### 2.4. Oxidative Stress Hypothesis of Alzheimer’s Disease

The oxidative stress hypothesis is based on oxidative stress reaction and the production of reactive oxygen species (ROS) [50]. Under AD pathological conditions, a large number of active metal ions, especially Cu, Zn and Fe, are imbalanced. When they bind to Aβ, they can catalyze the production of ROS [51]. In addition, in the cortex and hippocampus of AD patients, the catalytic activity of iron in cytochrome c (hydroxide reductase), which is involved in the mitochondrial energy transduction system, is decreased, resulting in the release of more superoxide anions and the decrease in antioxidant enzyme (catalase and glutathione peroxidase) activity [52]. Changes in mitochondrial function will cause electron leakage in the respiratory chain and bind to superoxide anions, thereby promoting ROS production [51,52]. The excessive production of ROS may damage the biochemical cascade reaction of neurons, worsen neural plasticity and accelerate the aging process [53]. Because the main source of ROS in cells is from mitochondria, excessive accumulation of ROS will lead to the destruction of mitochondrial homeostasis and mitochondrial dysfunction [54]. In addition, in the early stages of AD, ROS has been shown to cause Aβ deposition and tau hyperphosphorylation in the brain [41]. 

### 2.5. Neuroinflammatory Hypothesis of Alzheimer’s Disease

More and more research evidence shows that the development of AD is closely related to neuroinflammation [55]. In the early stage of AD, Aβ activates microglia by promoting the expression of pro-inflammatory cytokines such as tumor necrosis factor-α (TNF-α), IL-1β and IL-6 and then results in a neuroinflammatory environment to induce astrocyte activation and neuronal damage [56]. Neuron damage acts as damage-associated molecular patterns (DAMPs) [5], and in turn promotes the continuous activation of microglia and astrocytes to produce persistent chronic neuroinflammation. It leads to progressive neuronal apoptosis, accelerates brain damage and finally forms AD pathological characteristics [57].

Although Aβ plaque and NFT are the main pathological features of AD [58], persistent inflammatory response has been observed in the brain of most AD patients [59,60]. 

## 3. Neuroinflammation in Alzheimer’s Disease

### 3.1. Microglia and Astrocyte

**Figure 2:**
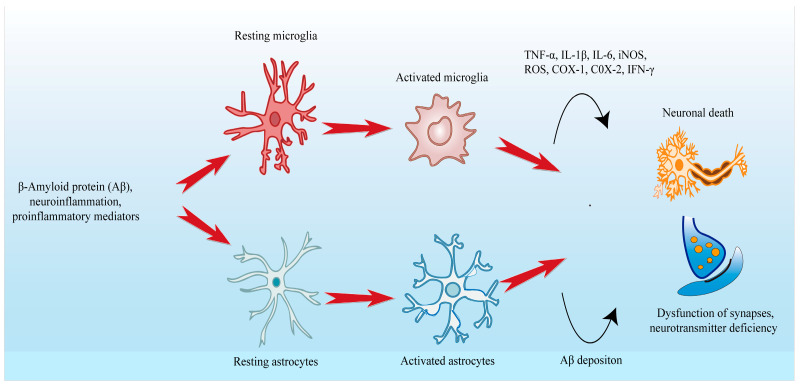
Mechanism of glial cells aggravating inflammatory response in brain neuroinflammation.

Microglia activation is the first sign of neuroinflammation [1,61]. Microglia are innate immune cells in the central nervous system and play a key role in the pathogenesis of neurological diseases [62,63,64]. Microglia express a divers set of pattern recognition receptors (PRRs) for Toll-like receptors (TLRs) and inflammasomes to monitor microbial invasion and nervous system damage in the central nervous system and regulate the brain microenvironment [65]. Previous studies have shown that microglia have two phenotypes: pro-inflammatory phenotype (M1) and anti-inflammatory phenotype (M2). M1 plays a pro-inflammatory role by secreting pro-inflammatory mediators, while M2 secretes anti-inflammatory mediators and nutritional factors to exert neuroprotective effects and remove apoptosis-related Aβ aggregation through phagocytosis [66,67]. In the early stage of AD, increased levels of Aβ, ATP and ROS lead to activation of the purinergic receptor P2X7 receptor (P2X7R) and down-regulation of myeloid cell trigger receptor 2 (TREM2), increasing calcium influx and, thus, activating microglia [56]. Furthermore, the BBB operates within the neurovascular unit (NVU). Under the pathological conditions of AD, leukocytes migrate through activated brain endothelial cells and penetrate the BBB, resulting in interacting with NVU components and then affecting their structural integrity and function [68]. BBB dysfunction affects Aβ clearance and endothelial cell transport by inducing peripheral immune cells such as neutrophils, monocytes and others to enter the brain and then release inflammatory cytokines to activate glial cells [69]. M2 microglia can clear Aβ through phagocytosis and prevent the formation of Aβ plates. Over time, the efficiency of Aβ clearance decreases and then results in the formation of extracellular Aβ plaques [56], when M2 microglia recognize pathogen-associated molecular patterns (PAMPs) or PRRs-induced DAMPs [70]. As shown in Figure 2, activated microglia are switched from the M2 to the M1 and eventually replaced by M1 in the later stages of brain injury [10]. Excessively activated M1 microglia release pro-inflammatory cytokines and oxidants, including inducible nitric oxide synthase (iNOS), ROS, interferon-γ (IFN-γ), TNF-α, cyclooxygenase 1 (COX-1), cyclooxygenase 2 (COX-2) and IL-1β [66]. Pro-inflammatory cytokines can induce immune response and transient inflammation to neutralize and eliminate toxic molecules and cell debris [71]. Under normal physiological conditions, microglia are rapidly switched from the M1 phenotype to the M2 phenotype and then secrete the appropriate nutritional and anti-inflammatory factors including brain-derived neurotrophic factor (BDNF), nerve growth factor (NGF) and interleukin-1 (IL-1) to terminate the immune response and inflammation [71,72]. However, under the pathological conditions of AD, it is difficult for microglia to go back the M2 phenotype [70], resulting in a serious imbalance between pro-inflammatory cytokines and neuroprotective factors, thus inducing chronic neuroinflammation. The production of inflammatory cytokines may reduce the expression of anti-apoptotic factor B-cell lymphoma/leukemia-2 gene (Bcl-2) and increase the expression of pro-apoptotic factors Bax and cysteinyl aspartate-specific proteinase-3 (caspase-3), which will initiate the caspase cascade reaction and lead to neuronal apoptosis [73,74]. 

Astrocytes are specialized glial cells, the most widely distributed cells in the mammalian brain, found in sebum and gray matter [75]. Astrocytes can promote synapse formation, maintain neurotransmitters and protect neurons, and respond as part of the brain’s immune response [61,76]. In addition, astrocytes play an important role in the occurrence of neuroinflammation and Aβ clearance. Pro-inflammatory cytokines secreted by microglia turn them into reactive astrocytes in AD [10]. Reactive astrocytes are a unique feature of the brain in AD patients. It loses the ability to protect neuronal survival and promote synapse formation and phagocytosis and releases pro-inflammatory factors that cause neuronal damage [67]. Astrocytes can mediate Aβ uptake and clearance through transporters and receptors such as low-density lipoprotein receptor-associated protein 1 (LRP1) and scavenger receptor class B member 1 (SCARB1); however, the Aβ clearance ability of reactive astrocytes decreased, causing Aβ aggregation [56], resulting in decreased Ach levels, which in turn leads to synaptic dysfunction.

### 3.2. NF-κB Signal Pathways in Neuroinflammation

**Figure 3:**
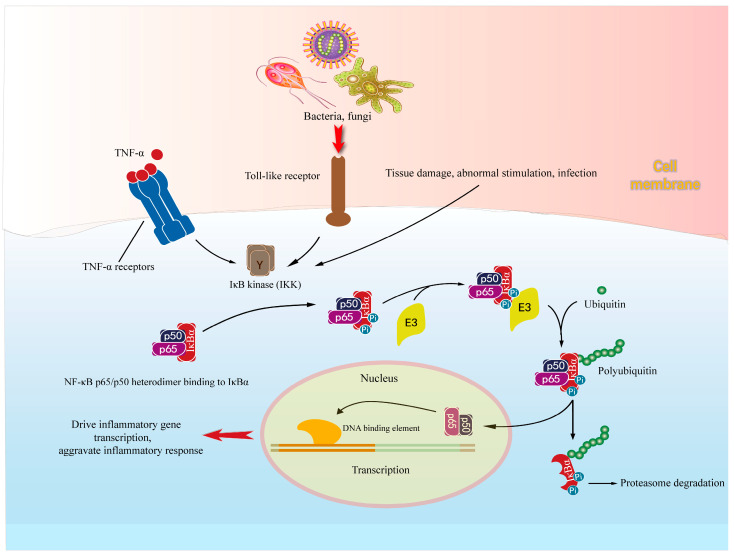
Activation and mechanism of NF-κB in neuroinflammation.

Neuroinflammation activates a large number of signal transduction proteins, by which the signal transduction cascades induce the activation of transcription factors to promote the production of pro-inflammatory cytokines, cytotoxic molecules and chemokines [1]. Among them, NF-κB is a key signaling molecule, because it can control the expression of many important pro-inflammatory molecules (such as iNOS, COX-2 and TNF-α) [77,78]. The activity of NF-κB depends on its nuclear translocation, which is associated with the inhibitory molecule IκBα [1]. Under steady-state conditions, the inhibitory molecule IκBα binds to the RelA/p50 heterodimer of NF-κB, resulting in the inactive form of NF-κB (Figure 3) [78]. Upon the stimulus by inflammatory mediators produced by microglia, IkB kinase (IKK) induces phosphorylation of IκBα, subsequently leading to its ubiquitination and finally proteasome degradation. As a result, RelA/p50 heterodimers can be translocated from the cytoplasm to the nucleus, and then connect to specific promoter elements and, thus, regulate the transcription of many inflammatory molecular genes [65].

## 4. Application Prospect of Anti-Inflammatory Activity of Natural Products in AD Treatment

Herbs and crude drugs in nature have therapeutic properties because they contain a large number of potential active ingredients, which are closely related to the study of human medicine [79,80]. In the case that existing drugs cannot cure AD, a large number of natural products have been reported to have therapeutic potentials for treating AD. Considering their rich nature resources and anti-inflammatory, antioxidant, anti-amyloid and anti-cholinesterase properties, their anti-inflammatory activity has attracted more and more attention for investigation of their application in the treatment of AD [23,67,81,82]. Usually, they are widely distributed in nature, easily available and less toxic [83]. They exert anti-inflammatory effects by mediating cell and signaling pathways associated with neuroinflammation, which may provide many excellent lead compounds for the development of anti-AD drugs.

## 5. Natural Products with Anti-Inflammatory Effects in Alzheimer’s Disease

### 5.1. Alkaloids

**Figure 4:**
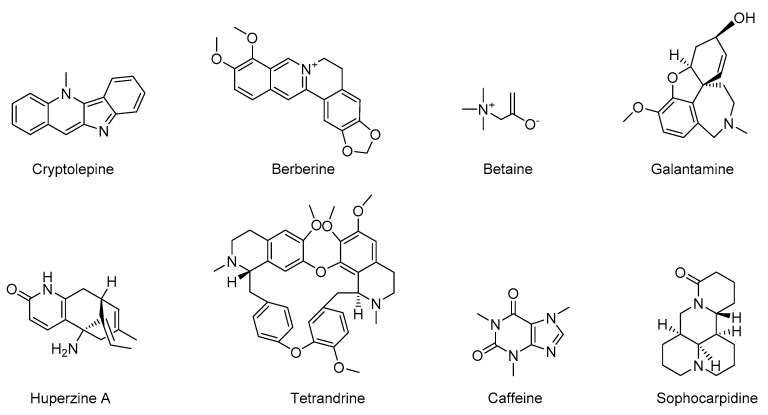
Chemical structure of natural alkaloids with anti-inflammatory activity.

Alkaloids belong to the secondary metabolites of organisms. They are a class of nitrogen-containing alkaline compounds that widely exist in nature. According to their chemical structure, they can be divided into indoles, carbazoles, carbolines, quinolines, isoquinolines, pyrroles, piperidines and purines [84]. Alkaloids usually have complex nitrogen-containing ring scaffolds with a variety of biological activities. The basicity of alkaloids is usually related to the hybridization of N-containing groups and may influence their biological activities. Their anti-inflammatory activity may provide neuroprotective effects in neurodegenerative diseases, so it provides a variety of hit compounds for developing new anti-inflammatory drug candidates to fight AD [85,86]. The structures of several alkaloids are shown in Figure 4. The following description is about their sources and their anti-inflammatory activity in the treatment of AD (Table 1).

Caffeine is a methyl xanthine alkaloid mainly found in *coffee* [87], which shows a preventive effect on AD with anti-inflammatory and anti-apoptotic properties [88]. Treatment with different concentrations of caffeine in lipopolysaccharide (LPS)-induced RAW264.7 cells showed that the expression of inflammatory mediators including nitric oxide (NO) and pro-inflammatory genes including COX-2, iNOS, IL-3, IL-6 and IL-12 were reduced and the signal transduction of phosphorylated p38MAPK was also inhibited. Moreover, the inhibition of LPS-induced NO production in zebrafish by caffeine was also observed [89]. Another study showed that caffeine intake significantly improved the performance of APPsw mice (a mouse model of AD) in Morris water maze, indicating its protective effect on cognitive impairment and improving memory [90]. Additionally, the treatment of caffeine could reduce Aβ deposition in the entorhinal cortex and hippocampus of APPsw mice [90,91]. Notably, caffeine has been found to cross the BBB due to its an intermediate lipophilicity but relatively high permeability [92].

Berberine, an isoquinoline alkaloid, is the main component of *Coptis chinensis*, a Ranunculaceae plant [93]. It shows promising neuroprotective effects in many neurodegenerative diseases [94]. It has been reported that berberine reduced the levels of pro-inflammatory cytokines COX-2, TNF-α and IL-1β in scopolamine-induced memory impairment and restored the levels of cAMP response element binding protein (CREB) and BDNF. Pretreatment of berberine in Aβ-induced mice could prevent the production of IL-6 and inhibit the expression of iNOS and COX-2 in primary microglia and BV2 cells [19]. In addition, berberine may antagonize Aβ-induced NF-κB activation in microglial inflammatory response by blocking the phosphatidylinositol 3-kinase (PI3K)/protein kinase B (AKT) and MAPK signaling pathways [95]. Another study found that the treatment of berberine reduced the activity of NF-κB-related transcription factors p50, p52, p65, c-Rel and RelB in the hippocampus of APP/PS1 mice and increased the activity of IκB protein, thereby inhibiting the activation of the NF-κB pathway and reducing neuroinflammation [96]. The pharmacokinetic study of berberine in rats showed that it could quickly pass through the BBB after 0.2 h of administration, reached the peak between 2 and 4 h and then was slowly metabolized [97].

Cryptolepine is an active ingredient in *Cryptolepis sanguinolenta* (blood red and white leaf rattan). It is an alkaloid of indoquinoline with significant anti-inflammatory activities [7]. Cryptolepine inhibited LPS-induced production of TNF-α, IL-6, IL-1β, NO, prostaglandin E2 (PGE2) and COX-2, and decreased iNOS protein level and mRNA levels in mouse microglia by partially targeting NF-κB signaling and attenuating p38/MAPKAPK2 phosphorylation [98]. Furthermore, cryptolepine could inhibit the expression of inflammatory mediators by blocking the binding of NF-κB to DNA after in vitro inflammatory stimulation [99].

Huperzine A is an alkaloid mainly extracted from *Huperzia serrata* [100]. It has a long history in treating dementia in China, mainly acting as an AChE inhibitor (AChEI) [101]. Huperzine A contains a tight tricyclic structure with an α-pyridone ring and a bicyclic [3.1.1] skeleton. The outer ring includes an ethylene moiety and a 3-carbon bridge with an α-NH_2_ group. This complex and unique molecular scaffold leads to its effective binding with AChE [102]. Additionally, huperzine A may interact with the nicotinic acetylcholine receptors (α7nAChRs and α4β2nAChRs) to reduce IL-1β, TNF-α expression and block the signal transduction of NF-κB, thereby inhibiting the transcription of inflammatory mediators and producing an effective anti-inflammatory effect [103]. The alkaloid-rich extract of Huperzia serrata can effectively inhibit the release of NO and inflammatory cytokines and the expression of iNOS and COX-2 by regulating the MAPK pathway in LPS-stimulated BV-2 microglia cells [104]. Huperzine A has been found to be an effective and reversible AChEI with the permeability of BBB [105].

Galantamine is an alkaloid derivative extracted from *Galanthus* [21]. It is a tetracyclic tertiary amine alkaloid with three chiral carbon atoms, mainly acting as an AChEI and also inhibiting inflammatory factors in activated microglia. Galantamine pretreatment can rescue brain injury by inhibiting generation of IL-1β and accumulation of activated microglia in neonatal hypoxic-ischemic (HI) rats [106]. Additionally, galantamine pretreatment can prevent the loss of neurons and astrocyte hypertrophy in HI rats. Galantamine can also increase the activity of the antioxidant enzyme catalase to eliminate excessive ROS induced by Aβ, thereby playing an antioxidant neuroprotective role with the permeability of the BBB [107,108]. 

Betaine is a quaternary ammonium alkaloid, which is a trimethyl derivative of glycine and widely exists in animals and plants. Among them, beet is one of the plants with the highest content of betaine [86]. Betaine could inhibit the activation of NF-κB and an inflammasome, nucleotide-binding oligomerization domain (NOD)-like receptor family pyrin domain containing 3 (NLRP3), by improving thioamino acid metabolism. Furthermore, betaine can reduce the secretion of inflammatory mediators, regulate energy metabolism and reduce cell apoptosis [86,109]. In vitro experiment results showed that betaine could inhibit the activation of NF-κB and release of inflammatory cytokines (TNF-α, IL-6, iNOS and COX-2) in LPS-induced RAW264.7 mouse macrophages [110]. Through various signaling pathways (NF-κB, NLRP3 and caspase-8/11), betaine could affect IL-1β processing and production mediated by classical and non-classical inflammasomes. Moreover, it can inhibit the release of IL-1β by reducing the shedding of IL-1β-containing membrane microbubbles and blocking the exocytosis of IL-1β-containing secretory lysosomes and exosomes [111]. However, it can pass through the BBB in a small amount, although betaine GABA transporter (BGT1) exists in the BBB [112].

Tetrandrine is a bisbenzylisoquinoline alkaloid isolated from *Stephania tetrandra* (mainly isolated from the roots and stems of Sinomenium actum Rehd) [7,113]. In the inflammatory model of glial cells, tetrandrine showed promising anti-inflammatory activities in Aβ-stimulated BV-2 microglia cells, by inhibiting the NF-κB pathway to inhibit the production of inflammatory cytokines [114,115]. It has also been reported that the treatment of tetrandrine can enhance dopamine receptor D2 (DRD2)-mediated nuclear translocation of astrocyte αB-crystallin through transcription activator 3 (STAT3), thereby inhibiting neuroinflammation with a good permeability of the BBB [116].

Sophocarpine comes from the dried roots and fruits of *Sophora flavescens*, a leguminous plant [117]. An in vitro study showed that sophocarpine pretreatment in Aβ-induced PC12 neuronal cells could prevent PC12 neuronal cell injury by inhibiting NF-κB nuclear translocation and reducing COX-2, PGE2 levels and iNOS expression [118]. Other studies have shown that sophocarpine treatment in APP/PS1 mice could reduce the production of pro-inflammatory cytokines by regulating inflammatory pathways, reducing Aβ plate deposition and improving cognitive dysfunction and brain injury [119].

**Table 1:** Types and main activities of alkaloids natural products.

Name	Species	Bioactivity	Reference
Caffeine	Methylxanthine alkaloids	Anti-inflammatory, anti-apoptosis, reduce Aβ deposition	[87,88,89,90,91]
Berberine	Isoquinoline alkaloid	Anti-inflammatory, neuroprotective	[19,93,94,95,96]
Cryptolepine	Indoloquinoline alkaloids	Anti-inflammatory	[7,98,99]
Huperzine A	New macrocyclic lycopodium alkaloids	Anti-inflammatory, AChEI	[101,103,104]
Galantamine	Phenanthrene alkaloids	Anti-inflammatory, reducing ROS, anti-oxidative stress, AChEI	[106,107]
Betaine	Quaternary-ammonium-type-alkaloid	Anti-inflammatory, anti-apoptosis	[86,109,110,111]
Tetrandrine	Bisbenzylisoquinoline alkaloid	Anti-inflammatory	[7,113,114,115,116]
Sophocarpine	Quinolizidine alkaloids	Anti-inflammatory, anti-apoptosis, reduce Aβ deposition	[118,119]

### 5.2. Flavonoids and Other Polyphenols

**Figure 5:**
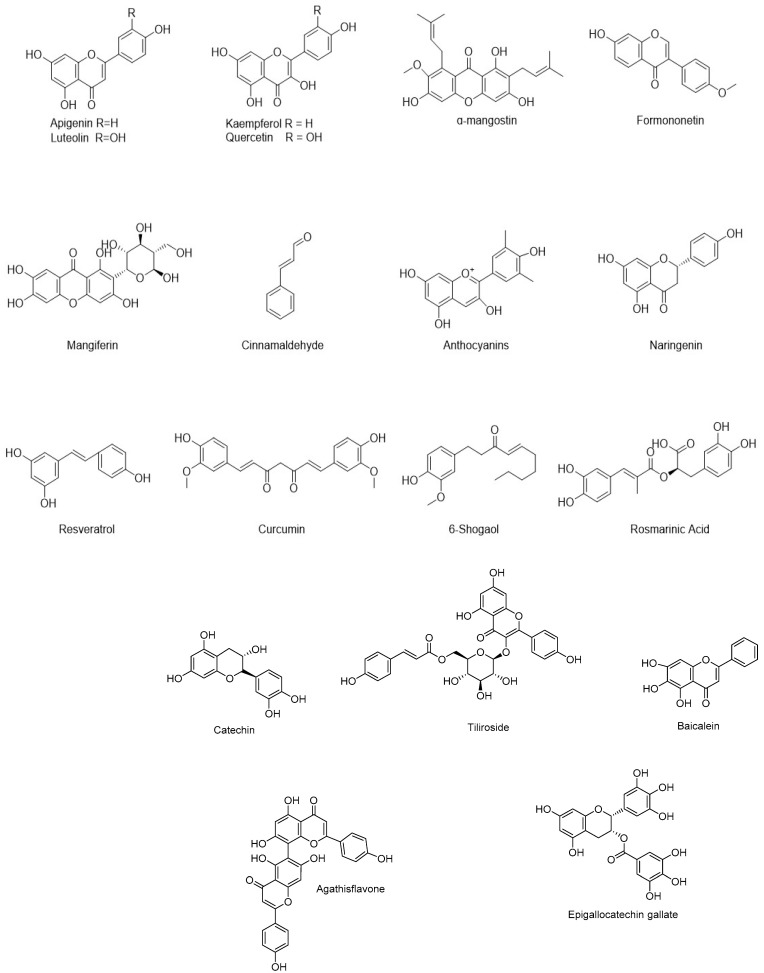
Chemical structure of natural flavonoids and other polyphenol with anti-inflammatory activity.

Flavonoids are naturally existing polyphenolic compounds and secondary metabolites of plants, especially abundant in vegetables and fruits [120]. As shown in Figure 5, many flavonoids and other polyphenols have demonstrated anti-neuritis activities [121], suggesting they may have therapeutic potentials for preventing the progression of AD [122,123].

Apigenin is a kind of flavonoid mainly isolated from *parsley, chamomile, celery,* and other plants of the Umbelliferae family [124]. It was also found that apigenin could pass through the BBB. Apigenin could reduce the expression of endothelial intercellular adhesion molecule-1 (ICAM-1) and vascular cell adhesion molecule-1 (VCAM-1) by inhibiting NF-κB to reduce the production of IL-1β, IL-6 and PGE2 [125]. In RAW264.7 cells, it was found that apigenin could significantly inhibit the production of NO, iNOS and COX-2 and the expression of cytokines (TNF-α, IL-1β, IL-6) by blocking the phosphorylation of MAPK signal molecules, including extracellular signal regulated kinase (ERK) and c-Jun N-terminal protein kinase (JNK) [126]. 

Luteolin belongs to the flavonoid family, originally found in some fruits and vegetables [127]. Luteolin can activate the microbiota–gut–brain axis after passing the blood–brain barrier, regulate systemic and cerebral insulin resistance and block Aβ deposition [128]. Furthermore, it inhibits IL-6, TNF-α and COX2 through the ERK/JNK/NF-κB pathway, thus reducing neuroinflammation [129]. Another study found that luteolin in Aβ1-42-induced mice significantly inhibited the phosphorylation of c-Jun N-terminal kinase (JNK)/p38 MAPK, attenuated microglia activation, reduced the production of pro-inflammatory cytokines and the accumulation of Aβ [73].

Curcumin from *curcuma* might be the most frequently studied natural polyphenol for the treatment of AD and its anti-inflammatory activity has been confirmed in multiple studies [7,21,130]. Curcumin binds to TLR and regulates downstream NF-κB, MAPK, AP-1, JAK/STAT and other signal pathways to inhibit inflammatory mediators such as IL-1β, IL-6, interlukin-8 (IL-8), TNF-α, iNOS and NO [131]. In BV-2 microglia stimulated by lipoteichoic acid (LTA) and LPS, curcumin could reduce the inflammatory mediators TNF-α, PGE2 and NO to inhibit neuroinflammation by blocking NF-κB and MAPK activation [132,133]. Many studies have tested the therapeutic effects of curcumin in Tg2576 transgenic mice (AD mouse model), which showed that curcumin could penetrate into the central nervous system to exert a wide range of anti-inflammatory effects by reducing oxidative stress and Aβ levels [134].

Cinnamaldehyde (CA) is a kind of flavonoid extracted from the bark of *cinnamon*, mainly responsible for the flavor and aroma of cinnamon [135]. It has been found that CA could bind to two cysteine residues in tau and, thus, prevent tau aggregation in vitro [136]. It was reported that the anti-inflammatory effect of CA was achieved in an mouse model of brain injury induced by ischemia/reperfusion by inhibiting the expression of signal transduction molecules such as Toll-like receptor 4 (TLR4), tumor necrosis receptor-related factor 6 and the nuclear translocation of NF-κB, thus reducing the level of pro-inflammatory factors (TNF-α and IL-1β) [67]. In addition, CA attenuated the activation of the NLRP3 inflammasome by inhibiting the expression of cathepsin B and P2X7R (P2 receptor) protein and reducing IL-1β and IL-8 during inflammation [137].

In RAW264.7 macrophages, α-mangostin (α-M), isolated from *Garcinia mangostana* L., could inhibit iNOS and COX-2 secretion by significantly blocking activation of the NF-κB and MAPK signaling pathway to reduce the release of inflammatory markers such as TNF- α, IL-1β, IL-6 and IL-8 [138]. α-M also helped to keep the tight connectivity of mouse brain microvascular endothelial cells 3 (bEnd.3), thus maintaining the morphological integrity of the BBB under neuroinflammation [139]. Moreover, the inhibition of NLRP3 activation might also be one of its anti-inflammatory mechanisms [140]. Other studies have found that α-M can inhibit LPS-induced TLR4 expression and NF-κB activation to attenuate neuroinflammatory response [141].

Formononetin is a member of flavonoid phytoestrogens, which is the main active ingredient of *red clover*, a leguminous plant [142]. In BV2 mouse microglia stimulated by LPS, formononetin significantly reduced TNF-α, IL-1β and IL-6 by inhibiting the NF-κB signal pathway and the production of COX2, PGE2 and iNOS [7,143]. In vivo, formononetin demonstrated a dose-dependent inhibition of TNF-α and IL-1β in the hippocampus of mice induced by high-fat diet. The most likely mechanism was to inhibit the pro-inflammatory NF-κB signal pathway and activate the anti-inflammatory Nrf-2/HO-1 signal pathway [143,144]. The disordered transport of Aβ across the BBB mediated by low-density lipoprotein-related protein 1 (LRP1) and the receptor for advanced glycation end products (RAGE) is a risk factor of AD pathogenesis. Formononetin treatment in APP/PS1 mice can promote LRP1-dependent Aβ clearance and inhibit the activation of the RAGE/NF-κB signaling pathway, thereby reducing the inflammatory response [145]. However, its capability to cross the BBB is poor. 

Baicalein is a natural flavonoid compound, which is the main bioactive component of *Scutellaria baicalensis Georgi* [146]. It has been found to have a neuroprotective role due to its anti-inflammatory activity in animal models [147]. In LPS-stimulated RAW264.7 cells, baicalein could inhibit iNOS, COX-2 and TNF-α in mRNA levels [148]. In microglia activated by LPS, baicalein could also inhibit NF-κB Nuclear translocation by blocking IκBα phosphorylation, thus significantly inhibiting the production of NO and the expression of iNOS protein. Furthermore, using baicalein to treat rotenone-exposed rats for a long time could improve their dyskinesia and reduce brain damage by inhibiting pro-inflammatory cytokines (TNF-α, IL-6) to regulate the activation of microglia and astrocytes, and blocking NF-κB and MAPK signal activation to down-regulate the TLR4 level in activated BV2 microglia [149]. A parallel artificial membrane permeability test to evaluate the ability of baicalein to pass through the BBB showed that baicalein could effectively pass through the BBB [150].

Naringin is a kind of citrus flavonoid with a variety of biological activities including significant anti-inflammatory activities [151]. Studies showed that naringin could inhibit the release of pro-inflammatory cytokine IL-1β and induce the expression of anti-inflammatory factor interleukin-10 (IL-10) and transforming growth factor β1(TGF-β1) [152]. Additionally, naringin could inhibit the NF-κB and MAPK signal cascade in inflammation and, thus, prevent the release of inflammatory mediators (TNF-α, IL-6, IFN-γ) [153]. The BDNF/CREB/tropomyosin receptor kinase B (TrkB) signaling pathway plays a key role in learning and memory. In the hippocampal tissue of an Aβ-induced model, naringin increased the level of BDNF and bound to TrkB, thereby improving synaptic plasticity and cognitive function. Since naringin could cross the BBB, naringin displayed the therapeutic potential to attenuate BDNF/TrkB/CREB signaling pathway-related neuroinflammation indued by Aβ [154]. 

Mangiferin (MGF) is a natural glucosyl flavone existing in the stem bark and leaves of *mango* plant (Mangifera indica) [7]. In LPS-stimulated BV2 microglia, MGF inhibited the activation of the NF-κB and NLRP3 inflammasome, thereby reducing production of IL-1β, IL-6, TNF-α, NO and the levels of iNOS and COX-2 [155,156]. The NLRP3 inflammasome can cause the activation of pro-IL-1β and the release of IL-1β. MGF might exert anti-inflammatory effects by inhibiting the activation of NLRP3 to activate caspase-1 and accumulate pro-IL-1β [156,157]. In addition, in APP/PS1 mouse models, MGF could regulate the activation of microglia and astrocytes in neuroinflammation and penetrate the BBB [156,158]. 

6-Shogaol is a phenolic phytochemical in *ginger*, which has been used as an anti-inflammatory drug in Asia for hundreds of years [21]. In the mouse model of cerebral injury induced by arterial occlusion (MCAO), 6-shogaol treatment significantly reduced the volume of cerebral infarction and the levels of malondialdehyde (MDA), ROS, IL-1β, TNF-α, COX-2 and iNOS by inhibiting NF-κB, ERK, JNK and p38 MAPK activation [159]. In scopolamine- and Aβ-induced mouse models, 6-shogaol improved cognitive impairment by inhibiting inflammatory mediators and increasing NGF levels [160]. Moreover, 6-shahaol showed promising therapeutic potentials for neurodegenerative diseases such as AD by inhibiting the release of inflammatory mediators and the activation of pro-inflammatory signal pathways in BV2, primary microglia and astrocytes [161]. By a parallel artificial membrane permeability test, 6-shogaol has been found that it could passively diffuse through the BBB [162].

Rosmarinic acid (RA) is a kind of polyphenol, which mainly exists in *Rosmarinus officinalis* [163]. It is reported that RA can inhibit hypoxia-inducible factor-1α (HIF-1α) and rescue neuronal damage induced by hypoxia-induced pro-inflammatory cytokines (TNF-α, IL-1β and caspase-3) [21]. RA could also improve Aβ1-42-induced neurotoxicity in an AD mouse model [164,165]. 

Anthocyanins are naturally occurring polyphenols, widely distributed in fruits and vegetables [166]. A study showed that Aronia dry extract (ADE) containing 25% anthocyanin could reduce the levels of inflammatory mediators (IL-1β, TNF-α, MDA) and lipid peroxides in vitro [167]. In LPS-stimulated RAW 264.7 macrophages, RCE (anthocyanin fraction of red clover extract) treatment could block the nuclear translocation of NF-κB subunit p65 and inhibit the expression of inflammatory mediators (IL-1β, TNF-α, Monocyte chemotactic protein 1 (MCP1), iNOS, COX-2) [168]. It was reported that a blueberry supplement rich in anthocyanins slowed an inflammatory response in primary microglia by inhibiting the activation of the p44/42 MAPK pathway and inhibited Aβ aggregation by increasing the Aβ clearance rate [169]. In addition, in the Aβ25-35-treated human neuroblastoma cell line (SK-N-SH), the pretreatment of anthocyanin inhibited the degradation of IκBα and the transfer of NF-κB p65 subunit from the cytoplasm to the nucleus, thereby reducing iNOS protein expression and NO production [170,171]. However, it was difficult for anthocyanins to cross the BBB into the brain after the evaluation of BBB permeability [172].

Catechin is an active polyphenol extracted from natural plant *tea*, which has good anti-inflammatory and antioxidant activities [173]. EGCG (epigallocatechin gallate) as one of the catechins is the most effective active ingredient in tea polyphenols [174]. EGCG can inhibit LPS-induced microglia activation and prevent inflammation-mediated neuronal damage [21]. Other research showed that EGCG could inhibit LPS-induced inflammatory responses including the production of NO and the expression of COX-2 and iNOS in BV2 microglia [175]. Furthermore, catechin could inhibit AChE activity and, thus, prevent Aβ aggregation [176,177]. Since EGCG easily penetrated the BBB, the clinical trials of phase 2 and phase 3 showed that the combination of EGCG and ascorbic acid could reduce neuroinflammation in AD patients [170].

Resveratrol (RSV) is a natural polyphenol mainly found in *red grapes* and *peanuts* [178]. RSV demonstrated excellent anti-inflammatory and antioxidant effects in animal models [2,130]. Previous studies have shown that RSV can inhibit the pro-inflammatory mediator, TNF-α, in microglia and promote the production of the anti-inflammatory molecule, IL-10, suggesting that RSV may have neuroprotective effects [21,179]. It has been reported that RSV may inhibit the apoptotic activity of p53 and forkhead box O (FOXO) by overexpressing SIRT1, assist neurons in resisting the release of ROS from the activated microglia by reducing the acetylation of NF-κB p65 and further inhibit the transcription of inflammatory mediators in neuroinflammation [5]. Other studies have shown that resveratrol may maintain the integrity of the BBB by reducing matrix metalloproteinases-9 (MMP9) to promote the elimination of Aβ deposition and, thus, attenuate glial neuroinflammation [180,181]. RSV can also cross the BBB [182].

Kaempferol is a flavonol, mainly isolated from the rhizome of *Kaempferia galanga* L., a ginger plant [183]. Research showed that kaempferol could inhibit neuroinflammation by inhibiting the NF-κB and p38 MAPK signaling pathway in LPS-induced BV2 microglia to reduce the production of pro-inflammatory mediators [184]. In addition, kaempferol 3-O-(2G-glucosylrutinoside)-7-O-glucoside (KGG) inhibited the production of iNOS, COX-2, NO, PGE2 and TNF-α in LPS-stimulated RAW 264.7 cells in a concentration-dependent manner. Western blotting also showed that KGG significantly enhanced the IκB protein expression level to inhibit NF-κB nuclear translocation and simultaneously inhibit the MAPKs and AKT signaling pathway [185]. In an in vitro BBB and intestinal drug permeability study, the results showed that kaempferol had good permeability of the blood–brain barrier [186].

Quercetin widely exists in the stem bark, flowers, leaves and fruits of many plants. Much research has shown that quercetin had strong anti-inflammatory activities [187]. Quercetin could inhibit the NF-κB pathway and induce the Nrf2/HO-1 pathway to mediate the reduction in iNOS and NO production in LPS-induced microglia cells [188]. Quercetin could also reduce neuroinflammation in the glial neuronal system induced by a neural toxin (1-methyl-4-phenylpyridine, MPP+). Additionally, it is found that the levels of inflammatory mediators are reduced by quercetin, thereby saving neuronal PC12 cells from extensive apoptosis [189]. Moreover, quercetin treatment in APP/PS1 mice could significantly reduce Aβ plaque, tau hyperphosphorylation and neuroinflammation to effectively improve their cognitive function [190]. Quercetin showed measurable in situ BBB permeability [191].

Agathisflavone is a flavonoid extracted from the Brazilian plant *Poincianella pyramidalis (Tul.)* [192]. It inhibits the NF-κB pathway in LPS-activated BV-2 microglial cells [7]. Tiliroside is a natural dietary glycoside flavonoid mainly found in Rosaceae and Malvaceae plants such as Tomczyk, Bazylko and Staszewska [193]. Its anti-inflammatory activity has been confirmed by many studies. At a certain concentration, tiliroside could effectively inhibit the release of pro-inflammatory mediators and the production of ROS in LPS-activated BV2 cells. Additionally, tiliroside could also inhibit the production of PGE2 and the expression of COX-2 [194,195]. It has been reported that tiliroside can improve neuroinflammation in LPS-induced BV2 cells by targeting the Nrf2 pathway and the NF-κB pathway to promote the expression of SIRT1 in microglia and deacetylate NF-κB subunits and, thus, inhibit the transcription of inflammatory genes [193].

### 5.3. Steroid Phytochemicals

**Figure 6:**
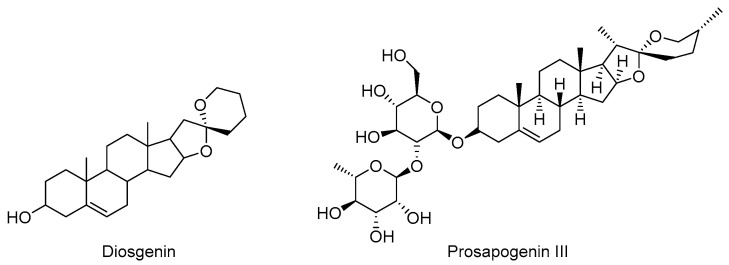
Chemical structure of natural steroids with anti-inflammatory activity.

Steroidal compounds are a large class of cyclopentadienyl phenanthrene derivatives widely distributed in nature. As shown in Figure 6, steroid compounds isolated from a variety of plants have demonstrated significant anti-inflammatory activities, suggesting that steroid compounds may have therapeutic potentials in the treatment of neurodegenerative diseases such as AD [196,197].

Diosgenin (DG) is a steroidal saponin widely found in *Rhizoma polygonati, Smilax china* and *Trigonella foenum-graecum* [198]. The anti-inflammatory activity of DG in the treatment of neurodegenerative diseases has attracted broad attention due to its few side effects [199]. It is reported that DG can inhibit the NF-κB pathway by regulating upstream receptors such as TLRs and downstream mediators including iNOS and COX-2 in LPS-stimulated RAW 264.7 macrophages [200,201]. Another study showed that DG could improve memory impairment in an AD model of Aβ1-42-stimulated mice by stimulating NGF to regulate the ACh-mediated cholinergic-anti-inflammatory pathway and also inhibiting the TLR/NF-κB pathway [202].

A study has shown that prosapogenin III can inhibit the phosphorylation of ERK1/2, JNK and p38 MAPK in LPS-stimulated RAW264.7 cells and the expression of some inflammatory mediators (IL-1β, IL-6, NO, iNOS and COX-2) by blocking the MAPKs/NF-κB pathway [203].

### 5.4. Terpenes

**Figure 7:**
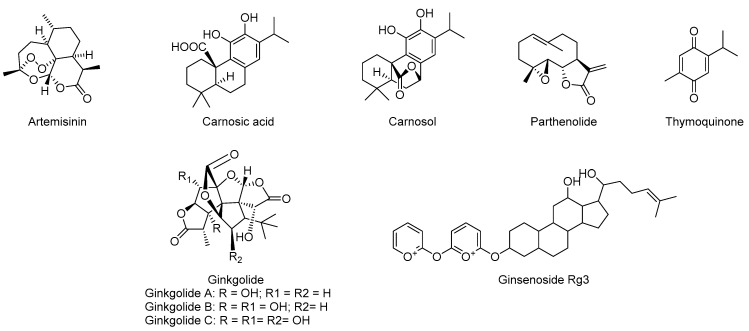
Chemical structure of natural terpenes with anti-inflammatory activity.

Terpenoids are one of the most abundant components of secondary metabolites produced by plants. As shown in Figure 7, the anti-inflammatory effects of many terpenes have been confirmed by many studies [204,205].

Artemisinin, as the most effective antimalarial drug, is a natural sesquiterpene lactone found in *Artemisia annua* L., which is a kind of lipid-soluble substance that easily penetrates the BBB into brain tissue [206]. Recently, this first-line clinical antimalarial drug has been proved to improve AD symptoms in animal models [207]. Previous reports showed that artemisinin could inhibit the release of IL-6, TNF-α, human macrophage chemotactic protein-1 (MCP-1) and NO in LPS-induced BV-2 microglia cells, and reduce IL-1β, IL-6 and TNF-α in hippocampus and cortex [7]. It was subsequently reported that artemisinin inhibited NF-κB directly or indirectly by silencing its upstream receptors, including ERK, JNK, PI3K/AKT and MAPK [208]. Further studies showed that artemisinin protected neurons by activating the ERK/CREB pathway and inhibiting the release of pro-inflammatory and apoptotic factors in an AD animal model of 3xTg mice [207]. However, its neurotoxicity may become an obstacle for further clinical research [208].

Parthenolide (PN) is a natural sesquiterpene lactone, which is the main active ingredient in the *chrysanthemum morifolium* with anti-inflammatory and neuroprotective potentials [209]. A study has shown that PN can reduce IL-6 and TNF-α levels in the hippocampus and cortex of rats [210]. In addition, in the rat model of intracranial hemorrhage (ICH), PN treatment inhibited the activation of the TLR4/NF-κB pathway, reduced the production of pro-inflammatory cytokines, alleviated the decrease in GSH level and SOD activity and inhibited the increase in ROS level [211].

Carnosic acid (CA) and carnosol are natural diterpenoids in *rosemary*, which have the permeability of the blood–brain barrier with neuroprotective potentials [7]. It has been reported that CA inhibits the activation of NLRP3 and NF-κB in LPS-induced RAW 264.7 macrophages, thereby reducing the production of inflammatory mediators such as TNF-α, IL-6 and NO [163]. Another study showed that CA can reduce the abnormal activation of Aβ-stimulated microglia and astrocytes in the brain of APP/PS1 mice. CA could improve Aβ deposition-induced neurodegeneration by inhibiting the CCAAT enhancer binding protein β (CEBP)-NFκB signaling pathway to reduce the production of IL-1β, IL-6 and TNF-α [212,213].

Ginkgolides are diterpenoids isolated from *Ginkgo biloba* leaves with potent anti-inflammatory and neuroprotective effects [214]. Its skeleton contains six rings, including three lactones, a tetrahydrofuran and a spiro [4.4] nonane carbocyclic ring. Different ginkgolides possess a difference in the number and position of hydroxyl groups on its scaffold [215]. Previous studies have shown that ginkgolides can enhance the expression of IκBα protein and reduce the expression of NF-κB p65 and Bax by inhibiting NF-κB to reduce the production of TNF-α and IL-1β in the AD model of APP/PS1 transfected HEK293 cell line (APP/PS1-HEK293) [216]. In addition, it has been reported that ginkgolide B (GB) can inactivate the NLRP3 inflammasome and reduce the level of pro-inflammatory cytokines by promoting autophagy degradation in LPS-stimulated BV2 cells, as well as in SAMP8 mice [217]. Interestingly, the relationship between GB and intestinal flora has been investigated. In an AD model of mouse induced by D-galactose and aluminum chloride, GB significantly reduced the levels of RAGE and Bax protein and then improved neuroinflammation through the flora–gut–brain axis by rescuing the decrease in Lactobacillus abundance and the increase in Bacteroides abundance [218]. However, ginkgolides usually have difficulty entering the brain through the BBB [219]. 

Ginsenoside Rg3 (GRg3) is the main active ingredient of *ginseng*. Since GRg3 is a small molecule, GRg3 can easily penetrate the BBB with high bioavailability [220]. It was reported that GRg3 significantly reduced the expression of TNF-α, IL-1β and COX-2 in the hippocampus of LPS-stimulated rats [221]. In addition, GRg3 could induce human microglia to switch from the M1 phenotype to the M2 phenotype by up-regulating acute cytokines (such as IL-10 and arginase 1), reducing the release of inflammatory factors while increasing the level of type A scavenger receptor (SRA) to promote the absorption and clearance of Aβ [222]. Another study showed that GRg3 effectively inhibited the binding of NF-κB p65 to DNA and the expression of TNF-α in Aβ42-induced BV-2 cells [223].

Thymoquinone (TQ) is the main active ingredient in *black grass* with promising therapeutic effects in AD models [224]. It has been reported that TQ can inhibit NF-κB-mediated neuroinflammation by activating the Nrf2/ARE signaling pathway and blocking the PI3K/Akt/NF-κB signaling pathway in LPS-stimulated BV-2 mouse microglia to reduce the release of inflammatory mediators [225]. Furthermore, studies have shown that TQ treatment significantly reduces plaque formation in the hippocampal CA1 region of male Wistar rat as AD models induced by hippocampal injection of Aβ, and, thus, protects neuronal cells from Aβ neurotoxicity [226,227]. In addition, in other AD models of D-galactose- and aluminum chloride-induced rat, TQ inhibited the TLRS receptor and its downstream signal NF-κB to reduce the production of TNF-α and IL-1β [228]. TQ has been found to be a promising chemotherapeutic compound against glioma and glioblastoma, suggesting it may cross the BBB smoothly [229].

## 6. Conclusions

With the aging of the population, AD has become an urgent issue for social public health, bringing a huge burden to individuals and society. A large number of studies have proposed various hypotheses on the etiology and pathological state of AD, providing valuable information for multi-target treatment of AD. Unfortunately, the current clinical use of anti-AD drugs can only delay the symptoms of AD patients, and cannot cure AD. The relationship between neuroinflammation and AD may provide a new strategy for discovery and development of novel agents to treat AD. Considering that natural products in general possess many meritorious properties for drug discovery and development, such as being diversely bioactive, less toxic, available and easily modified, anti-inflammatory natural products with the potential to prevent and alleviate AD symptoms, including alkaloids, steroids, terpenoids, flavonoids and polyphenols, have been summarized. However, more efforts on medicinal chemistry focused on natural products against neuroinflammation are expected to be made for the development of anti-AD drugs.

## Data Availability

Not applicable.
